# Cold water swimming reshapes gut microbiome to improve high-fat diet-induced obesity

**DOI:** 10.3389/fmicb.2025.1589902

**Published:** 2025-05-07

**Authors:** Jie Men, Chenglong Cui, Hao Li, Zhaowei Li, Yu Zhang, Zhiyu Liu, Qi Wang, Penghong Liu, Shuangling Zou, Zhengyang Yu, Yuxi Zhang, Simin Wu, Guoyu Zhu, Pengbo Wang, Xiaoli Huang

**Affiliations:** ^1^Department of Clinical Medicine, Fenyang College of Shanxi Medical University, Fenyang, China; ^2^Department of Basic Medical Sciences, Key Discipline of Fenyang College of Shanxi Medical University (Physiology), Fenyang, China; ^3^Department of Psychiatry, First Hospital of Shanxi Medical University, Taiyuan, China; ^4^Center for Translational Medicine, Key Laboratory of Birth Defects and Related Diseases of Women and Children (Sichuan University), Ministry of Education, West China Second University Hospital, Sichuan University, Chengdu, Sichuan, China; ^5^Longhua County Traditional Chinese Medicine Hospital, Chengde, China

**Keywords:** cold water swimming, gut microbiome, fecal microbiota transplantation, obesity, aerobic exercise

## Abstract

Hypothermia and swimming have been shown to alleviate high-fat diet (HFD)-induced obesity, with effects linked to the gut microbiota (GM). However, whether the effects of cold water swimming (CWS) on GM can be effectively transferred through fecal microbiota transplantation (FMT) has not been investigated. This study established mice models of obesity, CWS and FMT to investigate the mechanism by which CWS reshapes GM to improve HFD-induced obesity. Additionally, we analyzed the relationship between obesity phenotypes, GM composition, gene expression and CWS. The study found that HFD induced obesity phenotypes and GM dysbiosis in mice, while CWS produced opposite effects. The FMT results confirmed that CWS effectively alleviated HFD-induced lipid accumulation, metabolic disorders, and chronic inflammatory responses, which are associated with increased GM diversity, enrichment of beneficial bacteria, and the repair of intestinal barrier damage. Furthermore, these beneficial effects can be effectively transferred via FMT. The evidence from this study suggests that GM plays a critical role in the anti-obesity effects of CWS, with intestinal barrier repair emerging as a potential therapeutic target. This also provides scientific evidence for the feasibility of FMT as a strategy to combat obesity.

## Introduction

The global estimate for high body mass index (BMI ≥ 25kg/m^2^) indicates that ~2.63 billion individuals will be affected in 2023 and this number is projected to exceed 4 billion by 2035 (World obesity atlas, 2024; Nomura et al., 2024). High BMI is associated with the early onset of various non-communicable diseases, including type 2 diabetes, cardiovascular disease (CVD), cancer, and stroke (Piché et al., 2020). Obesity further exacerbates these risks. Currently, the primary approaches for treating obesity are medications, surgical treatment, physical activity, and dietary management (the significant role of the Mediterranean diet in obesity prevention and management should not be overlooked) (Dominguez et al., 2023). However, long-term use of medicine is often accompanied by adverse gastrointestinal reactions (Rubino et al., 2021; Sodhi et al., 2023), hypertension, and mental disorders (Gudzune and Kushner, 2024) and may even lead to significant reductions in heart weight and cardiomyocyte area (Martens et al., 2024). Surgical interventions carry risks of postoperative and metabolic complications (e.g., gastroesophageal reflux and severe nutrient deficiencies) as well as the need for re-operation (Perdomo et al., 2023). Notably, pharmacological and surgical interventions primarily address the obesity phenotype but do not positively impact obesity-induced declines in cardiopulmonary fitness, metabolic dysfunction, or mental disorders. Exercise, another effective intervention, is recommended by the American College of Cardiology (ACC) and the European Society of Cardiology (ESC). It should be emphasized that although exercise does not cause serious adverse effects or excessive disease burden, forms of exercise other than swimming may increase the risk of joint injuries in individuals due to high BMI (Roos and Arden, 2016).

Growing evidence indicates that gut microbiota (GM) is closely associated with diseases, including high-fat diet (HFD)-induced obesity (Perler et al., 2023). Meanwhile, GM is highly plastic and easily influenced by environmental factors, including diet and exercise. Differences in GM composition and function were observed between obese individuals and healthy subjects, and this finding was closely related to HFD-induced dyslipidemia and pro-inflammatory GM changes (Aron-Wisnewsky et al., 2021; Chambers et al., 2019). Clinical trials have shown that GM diversity and abundance of beneficial bacteria are significantly higher in individuals engaged in aerobic exercise than in obese individuals (Leong et al., 2020). Animal studies also support these findings, attributing the positive changes to GM remodeling (Dohnalova et al., 2022). Recent studies have found that low-temperature exposure induces dramatic changes in GM diversity, and composition, leading to weight loss (Ziȩtak et al., 2016), which is achieved by promoting fat browning, increasing energy expenditure, and repairing intestinal barrier damage (Li et al., 2019). Interestingly, these changes can be effectively transferred through fecal microbiota transplantation (FMT) (Chevalier et al., 2015), thus offering a novel option for obesity treatment.

In recent years, FMT has gradually attracted significant attention. Whether GM was transplanted from healthy mice to HFD-induced obese mice or from obesity-improved mice to healthy mice, the donor GM phenotype was consistently replicated (Barcena et al., 2019; Shtossel et al., 2023), emphasizing the causal relationship between FMT-mediated GM regulation and disease improvement. Moreover, although there is a correlation between GM donors and acceptors (Porcari et al., 2023), higher GM diversity and abundance in the donor are associated with an increased success rate of setting values when recipient conditions remain constant (Kump et al., 2018). Hence, the success of FMT engraftment is more influenced by the donor. Both exercise and hypothermia significantly enhanced GM diversity and abundance while demonstrating unique advantages in energy consumption. However, little is known about how to reshape GM based on Cold water swimming (CWS) to improve the mechanism of obesity.

To elucidate the relationship between CWS and HFD-induced iGM imbalance and to determine whether GM composition and changes are associated with the anti-obesity effects induced by long-term CWS. This study transplanted GM from mice subjected to long-term CWS into obese mice, validating GM as a key target for the an-ti-obesity effects of CWS, which also found that FMT effectively transferred the anti-obesity effects induced by CWS, including significant increases in GM diversity and abundance, as well as the repair of intestinal barrier damage and metabolic dysfunction. These findings lay the foundation for GM-targeted interventions to improve or treat obesity.

## Materials and methods

### Experimental animals and protocol

#### Experimental animals

This study used 46 SPF-grade male C57BL/6J mice aged 4 weeks, weighing 12–14 g, purchased from the Animal Research Center of Shanxi Medical University [SCXK (Shanxi) 2019-0004]. The laboratory conditions included a temperature of (25 ± 2°C), relative humidity (50 ± 5%), and a 12-h light-dark cycle. All mice were housed in open cages within a flexible membrane isolator with filtered air ventilation. Free access to food and water was provided, and after 1 week of acclimatization, the mice were randomly divided into the HFD group (*n* = 12), normal-fat diet (NFD) group (*n* = 12), CWS group (*n* = 12), and FMT group (*n* = 10). All animal experiments were approved by the Ethics Committee of Shanxi Medical University, Ethical Review Number: 2023020.

#### Experimental protocol

The NFD and CWS groups received standard chow (13.5% of calories from fat), while the HFD group was fed HFD (60% of calories from fat), provided by Book Yu Biological Technology (Shanghai) Co., Ltd., License No.: Su Feeding Permit (2019) 01008, and continued for 18 weeks. The CWS protocol was based on Da's study (Da Silva et al., 2020), with an adaptive CWS protocol (2 weeks): 3 days/week, 10 min/day, each session increased by 5 min; the temperature was 37°C, each reduction was 3°C; formal exercise protocol (16 weeks): 20°C, 30 min/day, 3 days/week, for a total of 16 weeks.

### Fecal microbiota transplantation

The FMT procedure was modified by Wang et al. (2020) and performed after the construction of the HFD and CWS models. Fresh feces from the donor (CWS group) were collected, homogenized in sterile phosphate buffer saline (PBS), and centrifuged at 4000 r/min for 5 min. The supernatant was aliquoted and stored at −80°C for further use. After 1 week of antibiotic pretreatment, the FMT was performed on the recipient (HFD group) with 200 μl per injection, three times per week until the end of the experiment. Feces from the FMT group were collected at the end of the study, snap-frozen in liquid nitrogen, and stored at −80°C for further analysis.

### Biochemical analysis

After modeling, the mice were fasted for 5 h and injected intraperitoneally with 25% urethane at a dose of 0.5 ml/100 g (20 g urethane in water, final volume 100 mL, 100 g mouse injected with 0.5 ml), and anesthesia was successfully achieved. Fresh blood samples were collected via cardiac puncture, centrifuged at 4000 r/min for 7 min, and the supernatant was used to measure serum biochemical parameters. According to the manufacturer's instructions, high-density lipoprotein (HDL), low-density lipoprotein (LDL), Total Cholesterol (TC), and Triglyceride (TG) were measured by colorimetric assays using an enzyme-linked immunosorbent assay (ELISA) kit purchased from Abbkine. Serum Lipopolysaccharide (LPS) levels were measured using a solid-phase sandwich ELISA kit purchased from Bio Swamp Ltd., following the manufacturer's instructions.

### Histological analysis

Tissue blocks (fat, liver, colon) from each mouse were collected, fixed in 4% para-formaldehyde solution for 36 h, and embedded in paraffin, followed by cutting into 4 μm slices. These slices were stained with Hematoxylin and Eosin (H&E) using standard techniques and then used for histopathological analysis. Observations were made under a light microscope to examine fat cell size in fat tissues, pathological changes in liver tissues the length of villi, and the number of goblet cells in the colon.

### Real-time fluorescent quantitative PCR (qrt-PCR)

Total RNA was isolated from adipose tissue and liver tissue, and quantitative qRT-PCR for mRNA was performed by adding a fluorescent substance to the qRT-PCR reaction system. The fluorescent signal was detected in real-time through binding to amplification products. The experimental procedure was as follows: First, RNA extraction was carried out, followed by reverse transcription, and then relative quantitative analysis of mRNA was performed. Information on tissue genes and the housekeeping gene GAPDH can be found in Table 1. Total RNA was isolated from the right lateral lobe of the mouse liver and adipose tissue, and qRT-PCR was performed as previously described. Based on the results of the aforementioned bioinformatics analysis, genes Uncoupling protein 1 (UCP-1), Peroxisome proliferator-activated receptor γ coactivator 1-alpha (PGC-1α), Protein kinase AMP-activated catalytic subunit alpha 1 (PRKAA1), and tumor necrosis factor-α (TNF-α) were selected for validation. The sample size was increased, and qRT-PCR technology was used to detect the expression levels of these genes in the NFD, HFD, CWS, and FMT groups. Their expression levels were measured. Total RNA was extracted using Buffer RLA, Buffer RW3, Buffer RW, and proteinase K. One microgram of total RNA from each sample was reverse transcribed using a reverse transcription system. Real-time amplification was performed using Master Mix and Primers. The initial stage was set at 95°C for 30 s, followed by a two-step program of 95°C for 10 s and 60°C for 30 s, cycled 40 times, and finally a three-step program of 95°C for 15 s, 60°C for 60 s, and 95°C for 15 s. The relative expression of genes was evaluated using the 2^∧^(–ΔΔCT) method.

**Table 1:** Gene organization and the reference gene GAPDH information.

**ID**	**Primer name**	**Sequence, 5^′^ → 3^′^**	**Base number**
UCP-1	UCP-1 F	AGGCTTCCAGTACCATTAGGT	21
	UCP-1 R	CTGAGTGAGGCAAAGCTGATTT	22
PGC-1α	PGC-1α F	TATGGAGTGACATAGAGTGTGCT	23
	PGC-1α R	CCACTTCAATCCACCCAGAAAG	22
PRKAA1	PRKAA1 F	GTCAAAGCCGACCCAATGATA	21
	PRKAA1 R	CGTACACGCAAATAATAGGGGTT	23
TNF-α	TNF-α F	GACGTGGAACTGGCAGAAGAG	21
	TNF-α R	TTGGTGGTTTGTGAGTGTGAG	21
GAPDH	GAPDH F	GTGTTCCTACCCCCAATGTGT	21
	GAPDH R	ATTGTCATACCAGGAAATGAGCTT	24

### Gut microbiota analysis

#### DNA extraction from samples

The total genomic DNA from GM was extracted according to the E.Z.N.A.^®^ soil DNA kit (Omega Bio-tek, Norcross, GA, U.S.), following the manufacturer's protocol. The quality of the extracted genomic DNA was checked using 1% agarose gel electrophoresis, and DNA concentration and purity were determined using NanoDrop2000 (Thermo Scientific, USA).

#### PCR amplification and sequencing library construction

Using the extracted DNA as a template, PCR was performed with the barcode-labeled forward primer 338F (5′-ACTCCTACGGGAGGCAGCAG-3′) and the reverse primer 806R (5′-GGACTACHVGGGTWTCTAAT-3′) targeting the V3-V4 variable regions of the 16S rRNA gene. Each sample was amplified in triplicates. After recovery and purification of the PCR products from the same sample, quantification was performed. The purified PCR products were then used to build sequencing libraries using the NEXTFLEX Rapid DNA-Seq Kit, and the original data was uploaded to the National Center for Biotechnology Information (NCBI) database under the sequence number PRJNA1203435.

#### Sequence data analysis

Quality control was performed on the paired-end raw sequencing sequences using fastp (Chen et al., 2018) (version 0.19.6). The FLASH (Magoc and Salzberg, 2011) (version 1.2.11) software was used for sequence merging. The sequencing analysis was based on the DADA2 (Bolyen et al., 2019) plugin in the Qiime2 pipeline (Callahan et al., 2016), where optimized sequences were denoised. The denoised sequences obtained from DADA2 were Amplicon Sequence Variants (ASVs). Mitochondrial and chloroplast sequences were removed from all samples. All samples were rarefied to equal sequencing depth, followed by taxonomic analysis at the ASVs level using a Naive Bayes classifier. Functional prediction analysis of the 16S rRNA sequences was performed using PICRUSt2 (Douglas et al., 2020) (version 2.2.0).

### Statistical analysis

Statistical analysis and plotting were performed using GraphPad Prism 10. If data met a normal distribution, means ± standard deviation were used to represent the data, and one-way analysis of variance (ANOVA) was used for intergroup comparisons. Non-normally distributed data were expressed as median (IQR), and group comparisons were performed using the Kruskal-Wallis test. Statistical significance was set at *P* < 0.05

All sequencing data analyses were conducted on the MegBio Cloud Platform, with the following details: Alpha diversity was calculated using the Mothur (Schloss, 2020) software, including Shannon and Chao indices and the Wilcoxon rank sum test was used for intergroup differences in Alpha diversity analysis. The similarity of GM structure among samples was tested using Principal co-ordinates analysis (PCoA) based on the Bray-Curtis distance algorithm and PERMANOVA non-parametric test was used to analyze significant differences in GM structure between sample groups. Linear discriminant analysis Effect Size (LEfSe) analysis (Segata et al., 2011) [Linear discriminant analysis (LDA) > 4, *P* < 0.05] was used to identify significant differences in bacterial taxa from phylum to genus level between groups. Distance-based redundancy analysis (db-RDA) was used to analyze the effects of CWS on GM structural changes. Linear regression analysis was used to assess the impact of CWS on GM alpha diversity indices in db-RDA. Species were selected for correlation network analysis based on Spearman correlation (|r| > 0.6, *P* < 0.05).

## Results

### CWS improves HFD-induced obesity phenotypes

**Figure 1:**
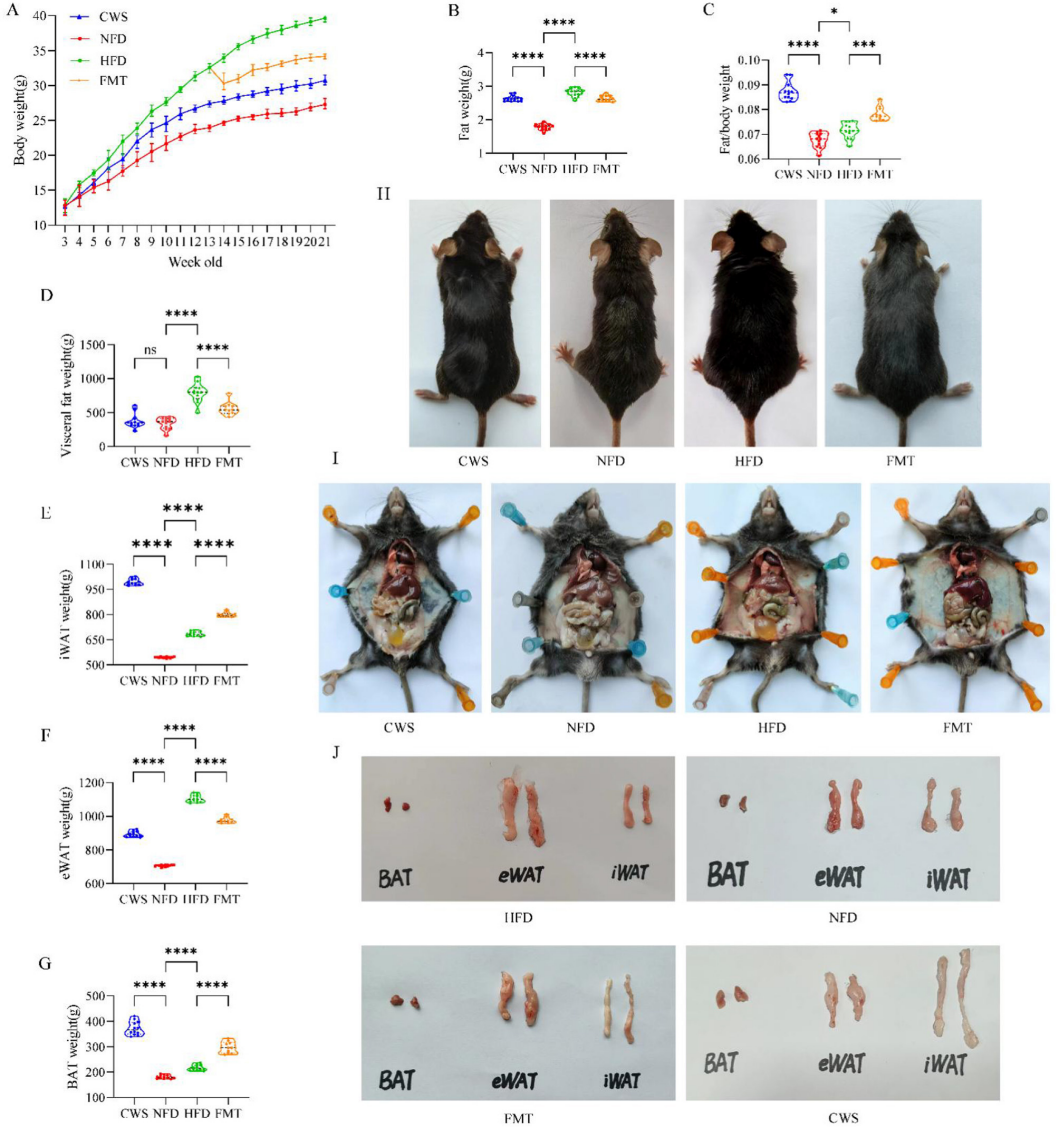
CWS improves the obese phenotype induced by HFD. **(A)** Changes in mice body weight during the experiment; **(B)** Total fat mass; **(C)** Fat/weight ratio; **(D)** Visceral fat; **(E)** iWAT; **(F)** eWAT; **(G)** BAT; **(H)** Mice morphology image; **(I)** Visceral fat image in the abdominal cavity; **(J)** Morphological image of fat types. Data are presented as mean ± SD; ns, no significant difference; *P < 0.05, **P < 0.01, ***P < 0.005, ****P < 0.001.

To evaluate the effect of CWS-based FMT on HFD-induced obesity, 10-week HFD-induced obesity and CWS mice models were established. Compared with the NFD group, mice in the CWS, and HFD groups showed significantly higher body weight (*P* < 0.01), consistent with changes in body size, with the HFD group exhibiting the most pronounced differences (Figure 1A). The total fat mass, visceral fat, and mice anatomy exhibited trends similar to those of body weight (Figures 1B, D, I). Notably, compared with the NFD group, the body weight-to-total fat ratio was significantly higher in the CWS and HFD groups, which can be attributed to weight gain induced by the HFD. However, the CWS group accumulated more fat, resulting in the highest body weight-to-total fat ratio. This suggested two mechanisms: on one hand, it shows that the compensation mechanism can obtain more energy from the physical objects consumed (Worthmann et al., 2017), and on the other hand, adaptive thermogenesis during CWS, which increased weight and fat mass in response to the low-temperature environment (Aldiss et al., 2022). To clarify the effects of CWS on mice fat, we analyzed fat types. Compared with the NFD group, epididymal white adipose tissue (eWAT) weight significantly increased in both the CWS and HFD groups (*P* < 0.001, Figure 1F). Inguinal white adipose tissue (iWAT) and brown adipose tissue (BAT) weights were significantly higher in the CWS group compared with the NFD group (*P* < 0.001, Figures 1E, G), indicating that CWS induces thermogenic regulation of energy homeostasis, leading to fat browning, a result consistent with previous studies (Cani and Van Hul, 2024). These findings suggested significant differences in obesity phenotypes between CWS and HFD models, but whether CWS-based FMT can improve HFD-induced obesity phenotypes remains unclear. Thus, after 8 weeks of CWS and HFD modeling, an FMT model was established. Remarkably, FMT replicated the CWS-improved obesity phenotype induced by HFD (*P* < 0.001, Figures 1A–G), suggesting that the GM altered by CWS held potential for obesity improvement.

### CWS improves HFD-induced metabolic disorders, browning pro-cesses, intestinal barrier function and gene expression

**Figure 2:**
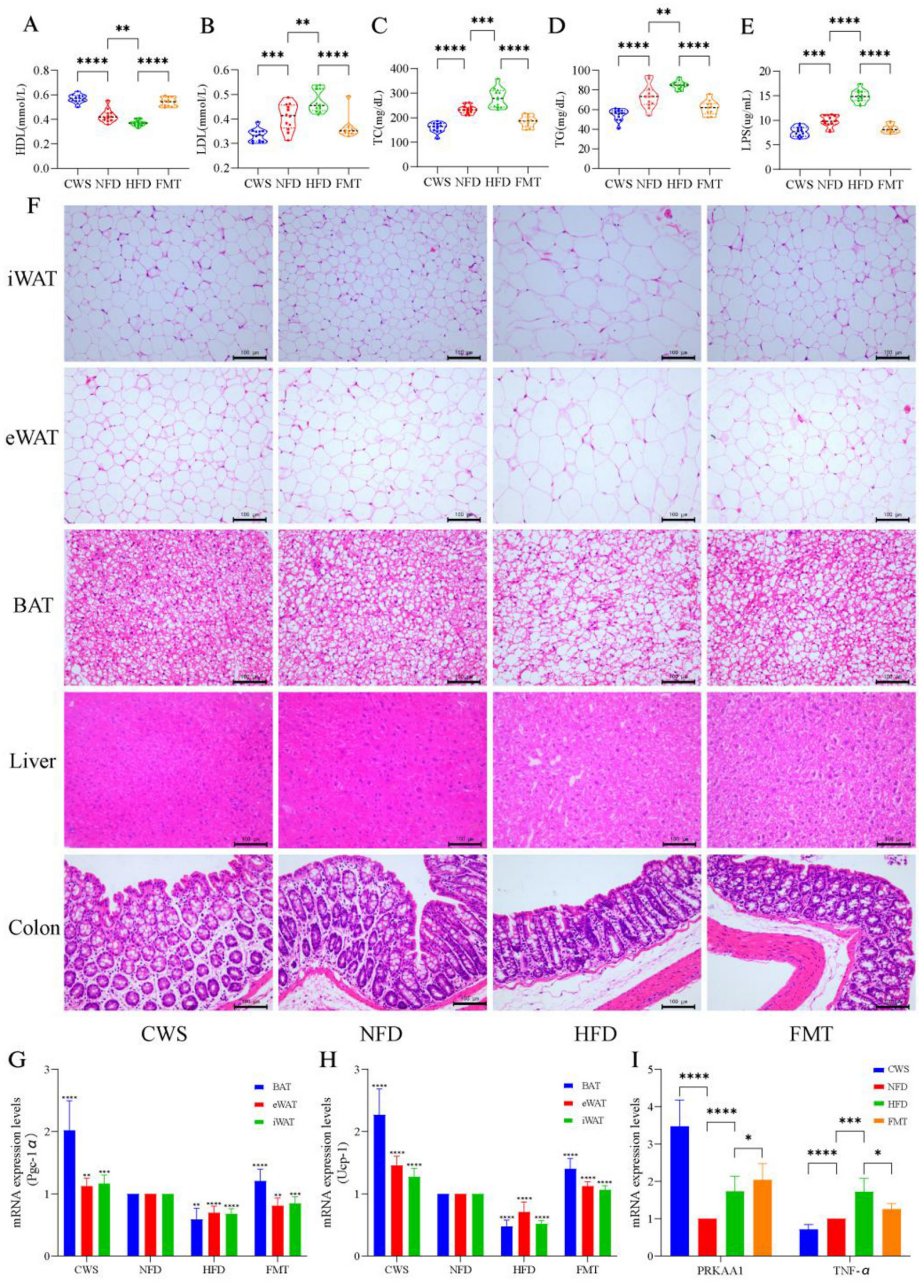
CWS improves HFD induced metabolic disturbances, browning processes, gut barrier function, and gene expression. **(A–E)** Impact on serum biochemical indicators: **(A)** HDL levels; **(B)** LDL levels; **(C)** TC levels; **(D)** TG levels; **(E)** LPS levels; **(F)** Representative images of fat and liver from H&E staining; **(G)** PGC-1α expression levels; **(H)** UCP-1 expression levels; **(I)** PRKAA1 and TNF-α mRNA abundance was determined by qRT-PCR analysis expression levels. Data are presented as mean ± SD; *P < 0.05, **P < 0.01, ***P < 0.005, ****P < 0.001.

Long-term CWS-induced adaptive changes positively affected dyslipidemia, inflammatory responses, fat browning, and gene expression. These changes could be replicated in recipients via FMT. To verify this possibility, we analyzed key lipid and inflammatory markers. Compared with the NFD group, HFD-induced mice exhibited dyslipidemia, with significantly elevated LDL, TC, and TG levels (*P* < 0.001, Figures 2B–D), reduced HDL levels (*P* < 0.001, Figure 2A), and increased LPS levels (*P* < 0.001, Figure 2E). The CWS group showed trends opposite to those of the HFD group, and the FMT group mirrored the CWS group's patterns. Combined with obesity phenotype data (Figure 1), these results demonstrated that HFD-induced weight gain was strongly correlated with dyslipidemia and inflammation, consistent with previous studies (Huang et al., 2021). To better understand the browning process of fat after CWS, pathological sections and H&E staining of adipose tissues and liver were conducted, and the H&E results were as expected. Compared with the NFD group, the HFD group exhibited significant expansion of eWAT cells and lipid accumulation in the liver (Figure 2F), whereas the CWS and FMT groups showed opposing results. BAT and iWAT contain higher mitochondrial density than eWAT, resulting in more efficient thermogenesis and metabolic benefits (Becher et al., 2021), consistent with the findings of this study. Compared with the NFD and HFD groups, the CWS group significantly inhibited adipocyte expansion and induced eWAT browning and BAT increases, as evidenced by differences in adipocyte size, number, and density (Figure 2F). These findings indicated that eWAT browning and BAT increases were more pronounced under dual stimuli of low-temperature exposure and exercise, which were significantly associated with improved HDL, LDL, TC, TG, and LPS levels. Notably, similar changes were observed in the FMT group, indicating that the GM phenotype reshaped by CWS could be effectively transferred via FMT.

HFD-induced dyslipidemia and inflammatory responses disrupted intestinal integrity and the intestinal barrier. Similarly, this study found that HFD-induced obesity resulted in a significant reduction in the number of goblet cells in the colon, along with decreased intestinal wall thickness and villus height, compared with the NFD group (Figure 2F). Dyslipidemia and increased LPS levels further supported evidence of intestinal integrity and barrier damage. Each intestinal cell contained up to 1,000 microvilli, structures that expanded the absorptive surface area by 25-fold. In contrast, the CWS group exhibited a marked increase in goblet cell numbers, intestinal wall thickness, and villus height (Figure 2F). These findings suggest that cold exposure enhanced intestinal absorptive capacity through these structural adaptations, and FMT effectively replicates these beneficial changes.

Low-temperature exposure activated UCP-1-mediated thermogenesis in BAT to counteract obesity. This study found that CWS significantly increased the expression of PGC-1α in various types of adipose tissue, with the highest increase observed in BAT (*P* < 0.001, Figure 2G). Similarly, the expression of UCP-1, which is regulated by PGC-1α, followed a similar trend (Figure 2H). This result aligned with fat browning induced by CWS and was linked to enhanced mitochondrial biogenesis and improved metabolism due to low-temperature exposure (Caesar et al., 2015). Another critical pathway involved in metabolism and inflammation was AMP-activated protein kinase (AMPK). The deletion of its PRKAA1 subunit can induce obesity and hepatic steatosis (Yang et al., 2022). Our findings revealed a significant increase in PRKAA1 expression in the CWS group compared with the NFD group (*P* < 0.001, Figure 2I), with similar changes observed in the FMT group. This indicates that CWS improves metabolism by inducing PRKAA1 expression, consistent with previous studies (Mulligan et al., 2007). Additionally, compared with the NFD group, the CWS group showed significantly inhibited TNF-α expression in the liver (*P* < 0.001, Figure 2I), whereas the HFD group exhibited a significant upregulation (*P* < 0.01, Figure 2I). After FMT, TNF-α levels were significantly reduced. In summary, long-term CWS ameliorated HFD-induced obesity by enhancing metabolism, promoting fat browning, increasing intestinal absorption, and modulating gene expression. These benefits were transferable through FMT.

### CWS alters gut microbiota diversity and community structure

**Figure 3:**
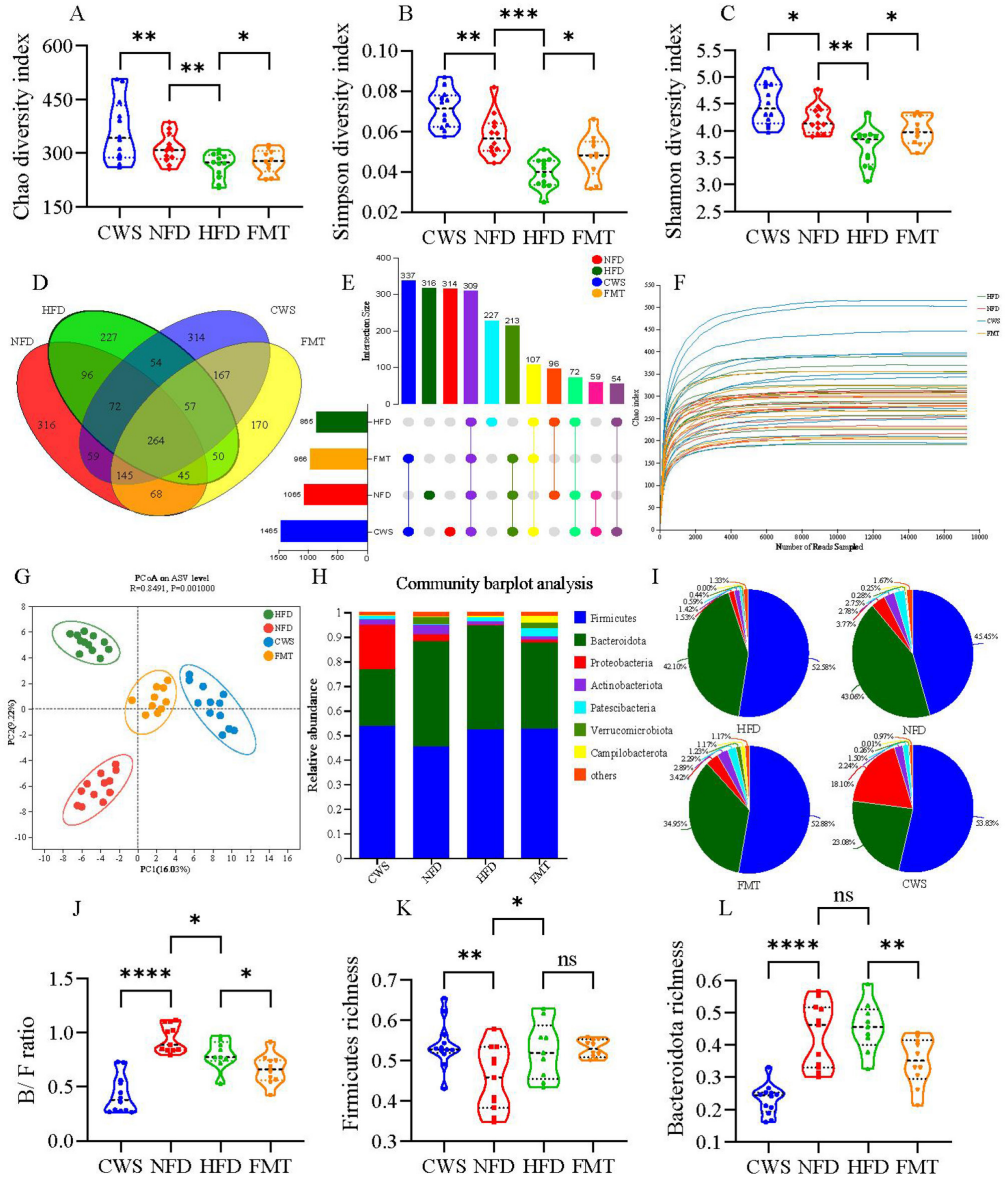
CWS alters GM diversity and community structure. **(A)** Chao index in α-diversity analysis; **(B)** Simpson index in α-diversity analysis; **(C)** Shannon index in α-diversity analysis; **(D)** Venn diagram of ASV levels; **(E)** UpSet plot of ASV levels; **(F)** Species rarefaction curve at Chao index level; **(G)** PCoA analysis of GM based on Bray_Curtis distance in feces. Principal components (PCs) 1 and 2 explained 16.03 and 9.22% of the variance, respectively; **(H)** Taxonomic composition of GM at the phylum level; **(I)** Pie chart of GM community at the phylum level; **(J)** B/F ratio; **(K, L)** Relative abundance of GM at the phylum level; **(K)** Firmicutes; **(L)** Bacteroidetes. Data are presented as mean ± SD; *P < 0.05, **P < 0.01, ***P < 0.005, ****P < 0.001.

Obesity is negatively correlated with GM diversity, and a decrease in GM diversity is consistent with previous studies observed in HFD mice (Wastyk et al., 2021). However, it remains unclear whether CWS and FMT can affect the GM diversity induced by HFD obesity. Fecal samples from the four mice groups were collected, and 16S rRNA gene sequencing was used to obtain 2,323,439 optimized sequences with 975,132,110 bases and an average sequence length of 420 bp. Alpha diversity results showed significant differences between groups; compared to the NFD group, the HFD group showed significant reductions in Chao, Simpson, and Shannon indices, while the CWS group showed significant increases (*P* < 0.05, Figures 3A–C). In comparison to the HFD group, the FMT group also showed significant increases. Venn diagrams (Figures 3D, E) and Chao index rarefaction curves (Figure 3F) further confirmed that HFD significantly reduced GM diversity, while CWS significantly increased GM diversity, and FMT could replicate the result of increasing GM diversity in the CWS group. Based on the Bray-Curtis distance, PCoA analysis visualized the differences in GM structure among the four groups (Figure 3G). PC1 and PC2 explained 16.03% and 9.22% of the variation, respectively, and the PCoA plot showed different clustering patterns: the HFD group clustered in the top left quadrant, the NFD group in the bottom left quadrant, while the CWS and FMT groups were clustered along the right axis. This indicates significant differences in the GM community structure among the HFD, NFD, CWS, and FMT groups, and the similarity of the GM community structure between the CWS and FMT groups further confirms the feasibility and efficacy of FMT. These results suggest that HFD significantly altered the GM community structure, while long-term CWS or FMT effectively prevents drastic changes in the GM community structure.

To further investigate the specific changes in GM, the relative abundance of GM at the phylum level was analyzed among different groups. Firmicutes and Bacteroidota were the dominant phyla, accounting for about 80% of the total sequences (Figures 3H, I). Compared to the NFD group, there was a significant 7.13% increase in Firmicutes abundance in the HFD group (*P* < 0.05, Figures 3H, I, K), consistent with previous studies (Zhang et al., 2020). However, CWS did not reverse this change. Compared to the NFD group, long-term CWS significantly reduced Bacteroidota abundance by 19.98%, while the FMT group showed a reduction of 8.11%. Similar trends were observed when comparing CWS and FMT to the HFD group (reductions of 19.02% and 7.15%, respectively). The consistent trend between the CWS and FMT groups suggests a relationship between long-term CWS and FMT. The ratio of Bacteroidota to Firmicutes (B/F ratio) was correlated with obesity; compared to the NFD group, the HFD group had a significantly lower B/F ratio (*P* < 0.05, Figure 3J). However, the trend in the CWS and FMT groups was inconsistent with the hypothesis, suggesting that the discrepancy might have been attributed to CWS and requires further verification.

### CWS alters GM composition

**Figure 4:**
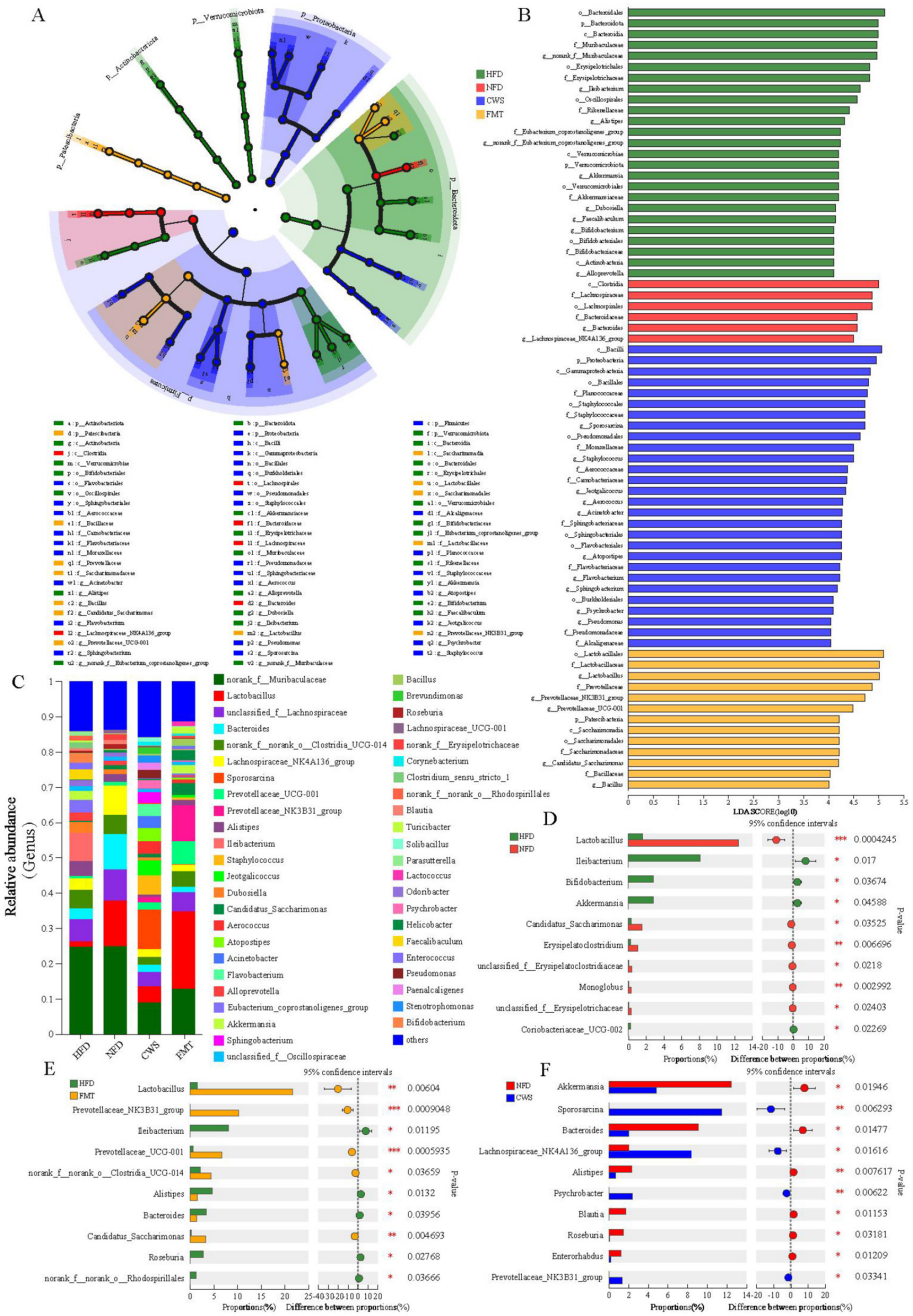
CWS alters the composition of GM. **(A)** LEfSe analysis of intergroup GM at the phylum-genus level Cladogram; **(B)** LDA at the phylum-genus level. (LDA >3 was considered to be the differential characteristic taxon); **(C)** Composition of GM at the genus level; **(D–F)** Dominant genera with significant differences between groups; **(D)** Comparison of HFD and NFD groups; **(E)** Comparison of HFD and FMT groups; **(F)** Comparison of CWS and NFD groups. Data are presented as mean ± SD; *P < 0.05, **P < 0.01, ***P < 0.001.

The obese phenotype induced by HFD is closely related to GM. In HFD mice, we observed increased lipid accumulation, metabolic endotoxemia (elevated blood lipid and inflammation), disrupted gut barrier integrity, and altered GM diversity and gene expression. These findings indicated that the composition of GM induced by HFD may have changed. To assess the effects of CWS, FMT, and HFD, we analyzed microbial composition at the phylum and genus levels. The LEfSe results indicated significant differences among bacterial taxonomic groups induced by CWS, HFD, and FMT, including phyla such as Firmicutes, Bacteroidota, Proteobacteria, Actinobacteria, Patescibacteria, and Verrucomicrobiota (Figure 4A). The LDA bar graph at the phylum-genus level showed 72 dominant genera, where CWS accounted for 38.89% (28 genera), HFD for 34.72% (25 genera), FMT for 18.05% (13 genera), and NFD for 8.33% (6 genera) (Figure 4B).

Further analysis at the genus level revealed significant differences among the dominant genera between groups (Figure 4C). Using the testing method for intergroup differences on the top 10 genera by relative abundance, we identified significant differences in genera between HFD and NFD groups, HFD and FMT groups, and NFD and CWS groups. Based on the community abundance data, a pairwise test method was used to analyze the top 10 genera with significant differences (*P* < 0.05). Harmful genera such as *Ileibacterium* and *Coriobacteriaceae_UCG-002* are significantly increased in the HFD group compared to the NFD group (Figure 4D). In the CWS group, beneficial genera such as *Lachnospiraceae_NK4A136_group, Prevotellaceae_NK3B31_group*, and *Sporosarcina* were significantly increased compared to the NFD group (Figure 4F). The changes in the relative abundance of these inflammation-related genera were consistent with changes in serum LPS levels. It is worth noting that in obese mice induced by a HFD, the abundance of beneficial bacteria such as *g_norank_f_norank_o_Clostridia_UCG-014, Lactobacillus, Prevotellaceae_NK3B31_group, Prevotellaceae_UCG-001*, and *Candidatus_Saccharimonas* significantly increased after FMT (Figure 4E). In summary, these results indicate that the differential changes in dominant microbial communities are consistent with the hypothesis of this study, that long-term regular CWS can reshape the GM composition in HFD-induced obese mice, and that this reshaping can be effectively transferred through FMT.

### CWS and HFD-induced GM changes correlate with metabolic indicators

**Figure 5:**
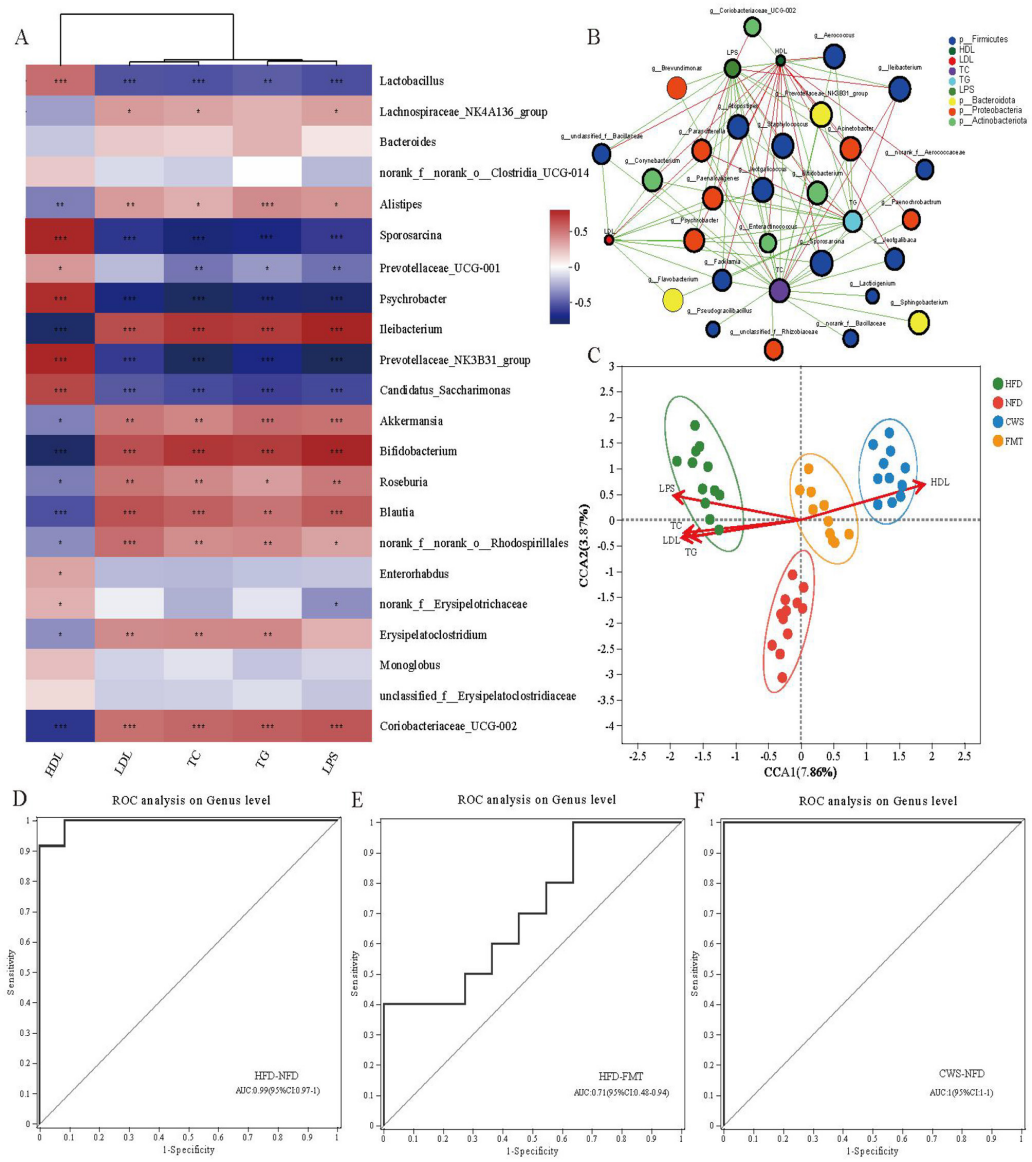
The changes in GM induced by CWS and HFD are correlated with metabolic indicators. **(A)** Correlation analysis of the top 22 dominant genera with metabolic indi-cators (red indicates positive correlation, blue indicates negative correlation); **(B)** Bipartite network plot of genera and metabolic indicators; **(C)** Ordination plot of species by RDA/CCA. CCA1 and 2 explained 7.86 and 3.87% of the variance, respectively.; **(D)** HFD and NFD related. Area Under Curve (AUC:0.99); **(E)** HFD and FMT related. (AUC:0.71); **(F)** CWS and NFD related. (AUC:1). ROC; *P < 0.05, **P < 0.01, ***P < 0.001.

At the genus level, a total of 22 bacterial genera with the highest abundance were selected, and a correlation matrix was generated using the Spearman correlation coefficient (Figure 5A). The potential relationship between GM changes and metabolic indicators caused by cryogenic swimming and HFD was clarified through correlation analysis. The results showed that most key bacterial abundances were highly correlated with metabolic indicators. Beneficial bacteria affected by CWS maintain intestinal integrity, increase Short-chain fatty acids (SCFAs) and improve blood lipids and inflammation, such as *Prevotellaceae_UCG-001, Prevotellaceae_NK3B31_group, Candidatus_Saccharimonas, Sporosarcina, Lactobacillus* and *Psychrobacter*, whose abundance is positively correlated with HDL, while LDL, TC, TG and LPS are negatively correlated. On the contrary, the abundance of harmful bacteria affected by HFD, *Alistipes, Ileibacterium*, and *Erysipelatoclostridium*, was significantly negatively correlated with HDL while positively correlated with LDL, TC, TG, and LPS. The results of the two-factor correlation network analysis between GM and metabolic indicators were consistent with the correlation analysis (Figure 5B). Meanwhile, TC and TG showed more bacterial genus correlations, followed by LPS, HDL, and LDL.

To further analyze the relationship between metabolic indicators and GM across different groups, Redundancy analysis/Canonical correspondence analysis (RDA/CCA) analysis was conducted (Figure 5C). The results showed that the sample distributions of each group were concentrated and significantly different. The microbiota functions of the CWS and FMT groups were similar, which was consistent with the PCoA results. The GM in the HFD group is highly correlated with TC, TG, LDL, and LPS, directly affecting the composition and function of GM in the HFD group. In contrast, the GM in the CWS and FMT groups is highly correlated with HDL, similarly affecting the composition and function of GM in the CWS and FMT groups. This difference is related to low temperature and HFD and is also consistent with the research hypothesis that CWS reshapes HFD-induced GM. In order to reveal the interrelationship between sensitivity and specificity among different groups, as well as the biomarkers at the genus level between different groups, a receiver operating characteristic (ROC) curve was constructed. The ROC curve analysis showed AUCs of 99%, 71%, and 100% respectively (Figures 5D–F). These results indicate that the changes in GM caused by CWS and HFD are highly correlated with metabolic indicators. The changes in GM in the FMT group are similar to those in the CWS group, providing further evidence to support the improvement of obesity by FMT.

## Discussion

Research indicates that weight loss in obese mice is associated with CWS-remodeled GM enhancing energy expenditure, but how CWS remodels GM in HFD-induced obese mice to ameliorate obesity, and whether this effect can be effectively transmitted through FMT remain unclear. This study systematically reveals for the first time that CWS alleviates HFD-induced obesity through multiple mechanisms, with evidence including the following aspects. First, a novel pattern of CWS remodeling GM to coordinate energy homeostasis. CWS activates the AMPK/SIRT1/PGC-1α signaling pathway to induce adipose browning, significantly improving energy metabolism in HFD mice, which synergizes with enhanced GM diversity and specific enrichment of anti-inflammatory and metabolism-regulating bacterial genera. Second, this study provides the first evidence that CWS induces increased intestinal barrier integrity and absorptive area in mice. CWS reduces LPS levels while significantly enriching intestinal barrier-repairing bacterial genera, markedly enhancing gut barrier function. Third, CWS-remodeled GM can be effectively transferred through FMT. Recipients post-FMT show high similarity in GM structure with donors, with a concurrent recapitulation of obesity phenotype improvement and intestinal barrier restoration.

Increasing evidence suggests that low-temperature exposure alleviates HFD-induced obesity and is associated with the reshaping of the GM (Worthmann et al., 2017; Ziȩtak et al., 2016). The results of our study showed that CWS, while increasing energy expenditure and reducing lipid accumulation, also induced significant changes in GM. GM diversity reflects the co-evolution between microbial communities and the host, which is consistent with the decrease in GM diversity caused by HFD and the increase due to CWS. Our study further revealed that CWS increases the proportions of the phyla Firmicutes and Bacteroidota. Overall, these results suggest that adaptive mechanisms in the host play a role in improving metabolism and controlling body weight. Both low-temperature exposure (Chevalier et al., 2015) and exercise (Cani et al., 2019) can modulate GM to facilitate fat browning (Pereira et al., 2022).

Mechanistically, CWS induces adipose browning and increases energy expenditure by activating UCP-1 and PGC-1α expression in BAT, ultimately reducing lipid accumulation (Liu et al., 2019). Through dual stimulation (cold exposure and exercise), CWS activated AMPK through dual stimulation (cold exposure + exercise) and catalyzed upregulation of subunit PRKAA1 expression (Figure 2I), which further promotes SIRT1 activity and leads to significant upregulation of PGC-1α, the core regulator of mitochondrial biosynthesis. This drove eWAT browning and BAT activation, manifested as a marked increase in iWAT and BAT mass (Figures 1E, G). Additionally, PGC-1α promoted mitochondrial thermogenesis and energy expenditure by elevating UCP-1 transcriptional levels, thereby reducing lipid accumulation (Figure 1F). These findings demonstrate that CWS likely promotes adipose browning via the AMPK/SIRT1/PGC-1α pathway, attributable to both exercise and cold exposure. This mechanism shares commonalities with metabolic improvements observed in cold exposure studies (Chevalier et al., 2015), but this study is the first to demonstrate that cold-water swimming synergizes with exercise adaptation through the AMPK/SIRT1/PGC-1α axis, achieving more pronounced adipose browning and metabolic benefits. Notably, FMT successfully transfers these adaptive changes. The data further support FMT as an effective strategy for ameliorating obesity.

HFD-induced GM imbalance led to a pro-inflammatory environment that accelerates metabolic disorders (Cai et al., 2024), while also disrupting intestinal barrier function and increasing intestinal permeability (Tulkens et al., 2020). These changes accelerate the release of LPS into the blood, inducing systemic chronic inflammation. A growing body of evidence suggests that impaired intestinal barrier function was associated with obesity (Beisner et al., 2021; Zhang et al., 2020). This study found that in HFD mice, CWS significantly reduced the levels of some bacteria associated with LPS and obesity, including *Ileibacterium, Coriobacteriaceae_UCG-002, Akkermansia*, and *Bifidobacterium*. On the contrary, the levels of beneficial bacteria that resist LPS and obesity were significantly increased, including *Lachnospiraceae_NK4A136_group, Prevotellaceae_NK3B31_group, Sporosarcina*, etc. This ultimately led to a decrease in LPS levels in the CWS group and FMT group. Correlation analysis showed that HFD-induced bacterial spectrum was positively correlated with LPS, while CWS could significantly inhibit the increase in LPS. An increase in the abundance of *Alistipes, Ileibacterium*, and *Erysipelatoclostridium* was observed in the HFD group, but a decrease in relative abundance in the CWS and FMT groups. LPS stimulated macrophages to produce TNF-α, which could further activate macrophages, forming a vicious two-way cycle, thereby exacerbating the inflammatory response (He et al., 2012). In this study, it was found that elevated levels of LPS are associated with a significant upregulation of TNF-α mRNA expression levels and were related to HFD induction, while CWS significantly improved this vicious cycle. The changes in intestinal barrier and permeability are the main reasons for the increased levels of LPS in the blood. Evidence of intestinal damage was observed in the HFD group, including a significant reduction in intestinal wall thickness, villus height, and the number of goblet cells, as well as a marked decrease in intestinal absorptive area. Conversely, the CWS group showed the opposite results. Interestingly, when the GM after CWS intervention was transplanted into obese mice, similar changes to the CWS group were observed. These changes helped the host adapt to low-temperature exposure and assisted in gut injury repair, consistent with previous research (Chevalier et al., 2015). Reports have indicated that low-temperature exposure increases LPS and TNF-a levels (Luo et al., 2019), and decreases GM diversity and beneficial bacterial abundance, which is inconsistent with this study. These studies mainly focus on single or short-term low-temperature exposure, whereas this study is based on long-term low-temperature exposure and includes swimming adaptation. Moreover, the subjects of this study are obese mice induced by HFD. The results of short-term low-temperature exposure suggest that changes in the GM are related to temperature and not related to the GM changes caused by obesity (Ziȩtak et al., 2016). This difference can also be further explained by the coordination of energy homeostasis in response to low-temperature exposure, which includes fat browning, energy intake, and environmental adaptation.

GM can be effectively transferred through FMT to treat diseases induced by GM imbalance due to HFD, including metabolic syndrome and obesity. An effective FMT setting is the key and prerequisite for remodeling host GM. On the one hand, it is influenced by the donor. Studies have shown that the success rate of receptor setting is related to the donor GM, and the difference ranges from 0 to 90% (Wang et al., 2024). This variability is primarily affected by the composition of the donor GM, including GM diversity and beneficial bacterial abundance (Porcari et al., 2023). A non-random cohort study found that an increase in the donor's GM abundance was correlated with the clinical success of FMT (Kump et al., 2018). Another systematic review of 25 studies evaluating FMT indicated that donor GM diversity is a predictive factor for treatment outcomes (Rees et al., 2022). On the other hand, the recipient's GM tolerance is another crucial factor for the success of FMT, with lower initial GM diversity in subjects associated with improved outcomes (Kootte et al., 2017). Population cohort studies further demonstrated the bidirectional relationship between donors and recipients (Shtossel et al., 2023), emphasizing that donor selection should be a priority for FMT. This study selected GM after low-temperature exposure and exercise as the donor, which had a positive impact on the recipient. To further validate the hypothesis of this study that gut barrier and GM reshaping are effective strategies for improving HFD-induced obesity, FMT was performed. The experimental results of this study support this hypothesis with evidence, including the suppression of dyslipidemia and LPS. Since dyslipidemia and LPS have a mutually vicious cycle relationship and form the pathological basis of obesity, their improvement is crucial for improving obesity. In the FMT group, improvements in dyslipidemia and inflammation were observed, which were consistent with the CWS group, indicating that GM from CWS successfully colonized in obese mice through FMT. Second, the reduction in LPS-induced gut barrier damage. The involvement of the gut barrier in various gastrointestinal diseases is significant (Di Tommaso et al., 2021), coupled with adverse events related to FMT, occurring only in subjects with gut barrier dysfunction (Marcella et al., 2021). Although LPS leakage and gut barrier damage were observed in the HFD-induced obese mice, no gut damage was found after FMT. Instead, evidence of gut barrier repair was found, including decreased LPS levels, increased goblet cell numbers, and increased villus height in the FMT group. Third, significant changes in GM composition were observed in the FMT group, particularly an increase in GM diversity and beneficial bacterial abundance. A growing body of evidence suggests that exercise improves colonic antioxidant (Almasi et al., 2023) and anti-inflammatory (Cook et al., 2016) status, thereby enhancing the function of the intestinal epithelium, repairing intestinal barrier damage, and reshaping GM to improve obesity (Shao et al., 2022). Convincing evidence from FMT indicates that reshaping of the intestinal barrier and GM are alternative targets for CWS to improve HFD-induced obesity.

GM dysbiosis is an independent risk factor for obesity. Although we confirmed that obesity alleviation is linked to CWS-remodeled GM and FMT, our study has certain limitations. First, experiments were conducted only in a single mouse strain, and differences in responses across genetic backgrounds were not evaluated. Second, the functional roles of specific microbiota (e.g., the *Lachnospiraceae NK4A136* and *Prevotellaceae NK3B31* groups) remain unvalidated due to the lack of monobacterial transplantation or metabolomic analyses. Third, molecular mechanisms rely solely on correlational data, and direct causal relationships require further verification using pathway inhibitors or gene knockout approaches. Fourth, the adaptability and safety of long-term FMT therapy require further assessment. Future research should fully leverage multi-omics approaches (e.g., metagenomics, metabolomics) to realize their pivotal role in diagnostic applications (Marascio et al., 2023). Additionally, experiments in models with diverse genetic backgrounds are required to clarify the functions of specific microbiota and molecular mechanisms, as well as to evaluate the safety of FMT. Furthermore, whether lifestyle interventions (e.g., the Mediterranean diet) and CWS exhibit synergistic/additive effects warrant further exploration, which would provide critical insights for clinical translation.

In summary, this study found that HFD-induced obese mice had specific GM features, including a reduction in GM diversity and an increased abundance of bacteria associated with pro-inflammatory and metabolic disorders. Conversely, CWS provided key evidence for improving HFD-induced obesity, and these positive effects were effectively transferred through FMT. This study shows that CWS improves lipid metabolism, and inflammatory responses by reshaping GM, thereby improving HFD-induced obesity, and that FMT may become an attractive treatment strategy for improving obesity.

## Data Availability

All data generated or analyzed during this study are included in this published article and the original data was uploaded to the NCBI database under the sequence number PRJNA1203435. All other raw data generated in this study are available upon request from the corresponding author.

## Glossary

HDF, High-fat diet; GM, Gut microbiota; FMT, Fecal microbiota transplantation; CWS, Cold water swimming; BMI, Body mass index; CVD, Cardiovascular disease; ACC, American College of Cardiology; ESC, European Society of Cardiology; NFD, Normal fat diet; PBS, Phosphate buffer saline; HDL, High-density lipoprotein; LDL, Low-density lipoprotein; TC, Total Cholesterol; TG, Triglyceride; LPS, Lipopolysaccharide; ELISA, Enzyme-linked immunosorbent assay; H&E, Hematoxylin and Eosin; qRT-PCR, Real-Time Fluorescent Quantitative; GAPDH, glyceraldehyde-3-phosphate dehydrogenase; UCP-1, Uncoupling protein 1; PGC-1α, Peroxisome proliferator-activated receptor γ coactivator 1-alpha; PRKAA1, Protein kinase AMP-activated catalytic subunit alpha 1; TNF-α, Tumor necrosis factor-α; NCBI, National Center for Biotechnology Information; ASVs, Amplicon Sequence Variants; PCoA, Principal co-ordinates analysis; LEfSe, Linear discriminant analysis Effect Size; db-RDA, Distance-based redundancy analysis; eWAT, Epididymal white adipose tissue; iWAT, Inguinal white adipose tissue; BAT, Brown adipose tissue; AMPK, AMP-activated protein kinase; LDA, Linear discriminant analysis; B/F ratio, The ratio of Bacteroidota to Firmicutes; SCFAs, Short-chain fatty acids; RDA/CCA, Redundancy analysis/Canonical correspondence analysis; ROC, Receiver operating characteristic.
